# Supplementary material for: The interactome of intact mitochondria by cross-linking mass spectrometry provides evidence for coexisting respiratory supercomplexes
Source: Mol Cell Proteomics. 2017 Dec 8;17(2):216–32. doi: 10.1074/mcp.RA117.000470 (PMC5795388; doi:10.1074/mcp.RA117.000470)
Supplement: Supplemental Data [file supp_RA117.000470_133922_0_supp_23978_fzffwf.zip › spectra_annotation/mito_DR_spectra_annotation/426-1-7-1-8-1.pdf]

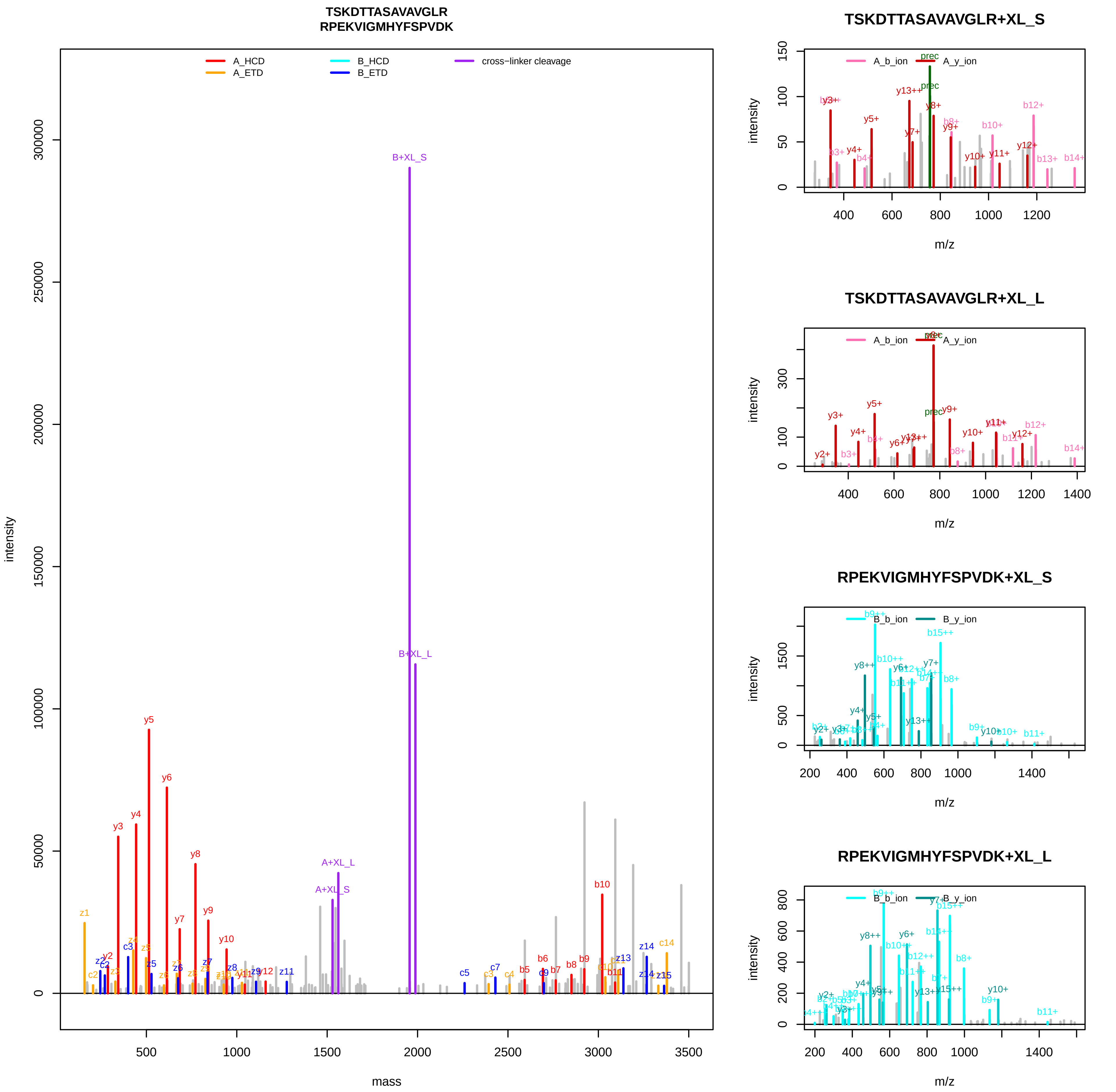
TSKDTTASAVAVGLR
RPEKVIGMHYFSPVDK
A_HCD
A_ETD
B_HCD
B_ETD
cross−linker cleavage
B+XL_S
B+XL_L
A+XL_L
A+XL_S
intensity
mass
0
50000
100000
150000
200000
250000
300000
500
1000
1500
2000
2500
3000
3500
y3
y4
y5
y6
y7
y8
y9
y10
y11
y12
z1
c2
c3
z5
z6
z7
z8
z9
z11
c5
c7
c3
c4
b5
b6
b7
b8
b9
b10
b11
z13
z14
z15
c14
TSKDTTASAVAVGLR+XL_S
TSKDTTASAVAVGLR+XL_L
RPEKVIGMHYFSPVDK+XL_S
RPEKVIGMHYFSPVDK+XL_L
A_b_ion
A_y_ion
B_b_ion
B_y_ion
prec
m/z
intensity
400
600
800
1000
1200
1400
200
0
50
100
150
300
500
1500
600
800
